# Puerperal Fever, as a Private Pestilence

**Published:** 1855-03

**Authors:** 


ART. V.—*Puerperal Fever, as a Private Pestilence.* By OLIVER WENDELL HOLMES, M. D., Parkman Professor of Anatomy and Physiology in Harvard University. Boston: Ticknor and Fields. 1855.

This is a pamphlet of sixty pages. Occasionally we become possessed of a book which provokes in us an inordinate desire to lend it, and this is one of them.

The body of this essay was read before a Boston medical society in 1843, and published in the New England Journal, a medical quarterly, which died at the end of its first year. Thus it happened that the essay was never largely read by the profession, though it was considered a "settler" in its day; having been adopted as authority by Copland, Ramsbotham, and the Registrar General of England, on the subject of the contagiousness of puerperal fever, and its transmissibility through the person of the medical attendant.

Dr. Holmes took the affirmative of the question, and supported the doctrine of contagion by an array of facts such as would upset all the theorizing in the world. After his essay it was no longer a question — the fact might be disputed, and so might the fact of the existence of such a man as Dr Holmes.

The recent teachings of Meigs on Childbed Fever, and the lectures of Prof. Hodge, of Philadelphia, have reopened the discussion, which Dr Holmes meets by the republication of his essay prefaced by an introductory.

The essay itself embodies all the evidence of contagion any rational mind could ask for: it is the introduction that gives spice to the pamphlet.

Dr. Meigs, in his late work, (about which we expressed some very decided opinions,) alludes to this essay as the "jejune and fizenless dreamings of sophomore writers." Dr. M. has an unfortunate monomania for voting himself *pontifex maximus* of Cloacina, and accordingly denouncing *ex cathedra* all opinions differing from his own. When a writer dares to dissent we hear forthwith of "long years spent in the practice of midwifery," and an absolute denial of any person's claim to a hearing. Dr. Holmes, however, has been considered as pretty good, and his puerperal statistics as still better, authority; and then, again, he holds a pen from which it is our devoutest wish to be spared when wielded in satire or anger.

We are particularly fortunate in this number. We have been severely cudgelled for a brace of sins against established opinion. One of these was a heretical departure from the canons of the Meigsian Midwifery; the other an infidelity on the saving efficacy of government schools. Just when tottering to defeat on the latter question, the Board of Visitors of the Michigan University came to our aid, by declaring in their official report, that our prophecies of political and ecclesiastical intermeddlings in the policy of such schools, has been fulfilled in Michigan; and now we are helped out, or sustained, in our first heresy, by the pen of Dr. Holmes: a pen which is rather a caustic pencil, leaving a deep sore wherever it touches.

We shall let Dr. Holmes write his own review, by sundry pithy quotatio from his introduction. And first, his advice to the disciples of the Jefferson school:

"To the Medical Students, into whose hands this Essay may fall, some words of introduction may be appropriate, and, perhaps to a small number of them, necessary. There are some among them who, from youth, or want of training, are easily bewildered and confused in any conflict of opinions into which their studies lead them. They are liable to lose sight of the main question in collateral issues, and to be run away with by suggestive speculations. They confound belief with evidence, often trusting the first because it is expressed with energy, and slighting the latter because it is calm and unimpassioned. They are not satisfied with proof; they cannot believe a point is settled, so long as every body is not silenced. They have not learned that error is got out of the minds that cherish it, as the tænia is removed from the body, one joint, or a few joints at a time, for the most part, rarely the whole evil at once. They naturally have faith in their instructors, turning to them for truth, and taking what they may choose to give them; babes in knowledge, not yet able to tell the breast from the bottle, pumping away for the milk of truth at all that offers, were it nothing better than a Professor's shrivelled forefinger."

Closely following this, the question is opened:

"Two widely known and highly esteemed practitioners, Professors in two of the largest Medical Schools of the Union, teaching the branch of art which includes the Diseases of Women, and therefore speaking with authority; addressing in their lectures and printed publications large numbers of young men, many of them in the tenderest immaturity of knowledge, have recently taken ground in a formal way against the doctrine maintained in this paper.* The first of the two publications, Dr. Hodge's Lecture, while its theoretical considerations and negative experience do not seem to me to require any further notice than such as lay ready for them in my Essay, written long before, is, I am pleased to say, unobjectionable in tone and language, and may be read without offence.

---

*On the Non-Contagious Character of Puerperal Fever: An Introductory Lecture. By Hugh L. Hodge, M. D., Professor of Obstetrics in the University of Pennsylvania. Delivered Monday, October 11, 1852. Philadelphia: 1852.

On the Nature, Signs and Treatment of Childbed Fevers: in a Series of Letters Addressed to the Students of his Class. By Charles D. Meigs, M. D., Professor of Midwifery and the Diseases of Women and Children in Jefferson Medical College, Philadelphia, etc., etc. Philadelphia: 1854. Letter VI.

"This can hardly be said of the chapter of Dr. Meigs' volume which treats of Contagion in Childbed Fever. There are expressions used in it which might well put a stop to all scientific discussions, were they to form the current coin in our exchange of opinions. I leave the 'very young gentlemen,' whose careful expositions of the results of practice in more than six thousand cases, are characterized as 'the jejune and fizenless dreamings of sophomore writers,' to the sympathies of those 'dear young friends,' and 'dear young gentlemen,' who will judge how much to value their instructor's counsel to think for themselves, knowing what they are to expect if they happen not to think as he does.

"One unpalatable expression, I suppose the laws of construction oblige me to appropriate to myself, as my reward for a certain amount of labor bestowed on the investigation of a very important question of evidence, and a statement of my own practical conclusions, I take no offence and attempt no retort. No man makes a quarrel with me over the counterpane that covers a mother, with her new-born infant at her breast! There is no epithet in the vocabulary of slight and sarcasm that can reach my personal sensibilities in such a controversy. Only just so far as a disrespectful phrase may turn the student aside from the examination of the evidence, by discrediting or dishonoring the witness, does it call for any word of notice.

"I appeal from the disparaging language by which the Professor in the Jefferson School of Philadelphia would dispose of my claims to be listened to. I appeal, not to the vote of the Society for Medical Improvement, although this was an unusual evidence of interest in the paper in question, for it was a vote passed among my own townsmen; nor to the opinion of any American, for none know better than the Professors in the great Schools of Philadelphia how cheaply the praise of native contemporary criticism is obtained. I appeal to the recorded opinions of those whom I do not know, and who do not know me, nor care for me, except for the truth that I may have uttered; to Copland, in his Medical Dictionary, who has spoken of my essay in phrases to which the pamphlets of American 'scribblers' are seldom used from European authorities; to Ramsbotham, whose compendious eulogy is all that self-love could ask; to the Fifth Annual Report of the Registrar General of England, in which the second-hand abstract of my essay figures largely, and not without favorable comment, in an important appended paper. These testimonies, half forgotten until this circumstance recalled them, are dragged into the light, not in a paroxysm of vanity, but to show that there may be food for thought in the small pamphlet which the Philadelphia Teacher treats so lightly. They were at least unsought for, and would never have been proclaimed but for the sake of securing the privilege of a decent and unprejudiced hearing."

Dr. Meigs supports his position by asserting that *only* parturient females are liable to this contagion: a good argument if true, but which suffers under analysis.

"The following statement is made by Dr. Meigs in his 142d paragraph, and developed more at length, with rhetorical amplifications, in the 134th. 'No human being, save a pregnant or parturient woman, is susceptible to the poison.' This statement is wholly incorrect, as I am sorry to have to point out to a Teacher in Dr. Meigs' position. I do not object to the erudition which quotes Willis and Fernelius, the last of whom was pleasantly said to have 'preserved the dregs of the Arabs in the honey of his Latinity.' But I could wish that more modern authorities had not been overlooked. On this point, for instance, among the numerous facts disproving the statement, the American Journal of Medical Sciences, published not far from his lecture-room, would have presented him with a respectable catalogue of such cases. Thus he might refer to Mr. Storrs' paper 'On the Contagious Effects of Puerperal Fever on the Male Subject; or on Persons not Childbearing,' (Jan., 1846,) or to Dr. Reid's case, (April, 1846,) or to Dr. Barron's statement of the children's dying of peritonitis in an epidemic of puerperal fever at the Philadelphia Hospital, (Oct., 1842,) or to various instances cited in Dr. Kneeland's article, (April, 1846.) Or if he would have referred to the New York Journal, he might have seen Prof. Austin Flint's cases. Or, if he had honored my essay so far, he might have found striking instances of the same kind in the first of the new series of cases there reported and elsewhere. I do not see the bearing of his proposition, if it were true. But it is one of those assertions that falls in a moment before a slight examination of the facts; and I confess my surprise, that a Professor who lectures on the Diseases of Women should have ventured to make it."

Dr. Holmes closes with a manly and energetic statement of the importance of the question, which does credit to his heart as well as his reason. We make no apology for our copious extracts. The subject is one of the last importance to the lives of parturient females, and to the consciences of practitioners.

"The teachings of the two Professors in the great schools of Philadelphia are sure to be listened to, not only by their immediate pupils, but by the profession at large. I am too much in earnest for either humility or vanity, but I do entreat those who hold the keys of life and death, to listen to me also for this once. I ask no personal favor; but I beg to be heard, in behalf of the women whose lives are at stake, until some stronger voice shall plead for them.

"I trust that I have made the issue perfectly distinct and intelligible. And let it be remembered that this is no subject to be smoothed over by nicely adjusted phrases of half-assent and half-censure divided between the parties. The balance must be struck boldly and the result declared plainly. If I have been hasty, presumptuous, ill-informed, illogical; if my array of facts means nothing; if there is no reason for any caution in the view of these facts; let me be told so, on such authority that I must believe it, and I will be silent henceforth, recognizing that my mind is in a state of disorganization. If the doctrine I have maintained is a mournful truth; if to disbelieve it, and to practice on this belief, and to teach others so to disbelieve and practice, is to carry desolation, and to charter others to carry it, into confiding families, let it be proclaimed as plainly what is to be thought of the teachings of those who sneer at the alleged dangers, and scout the very idea of precaution. Let it be remembered that *persons* are nothing in this matter; better that twenty pamphleteers should be silenced, or as many professors unseated, than that one mother's life should be taken. There is no quarrel here between men, but there is deadly incompatibility and exterminating warfare between doctrines. *Coincidences*, meaning nothing, though a man have a monopoly of the disease for weeks or months; or *cause and effect*, the cause being in some way connected with the person; this is the question. If I am wrong, let me be put down by such a rebuke as no rash declaimer has received since there has been a public opinion in the medical profession of America; if I am right, let doctrines which lead to professional homicide be no longer taught from the chairs of those two great institutions. Indifference will not do here; our journalists and committees have no right to take up their pages with minute anatomy and tediously detailed cases, while it is a question whether or not the 'black-death' of child-bed is to be scattered broadcast by the agency of the mother's friend and adviser. Let the men who mould opinions look to it; if there is any voluntary blindness, any interested oversight, any culpable negligence, even, in such a matter, and the facts shall reach the public ear; the pestilence-carrier of the lying-in-chamber must look to God for pardon, for man will never forgive him."

