# Correction

**Published:** 1855-04

**Authors:** 


*Correction.*—In our last number, having occasion to speak of a distinguished professor of midwifery, we mentioned him as "voting himself Pontifex Maximus of *Cloacina*." A few days since, we received a letter from a professional friend in the far east, (down in the neighborhood of sunrise,) complimenting us on sundry points, and among the rest on "the appropriate and purely classical allusion" above mentioned. We pocketed the letter and the compliment together, going our way with a pleasant feeling, as if we had been tickled and scratched in the most felicitous manner. "It is something," we said to ourselves, "to have a delicate classicality appreciated, in these days when learned allusion, or mythological metaphor, drop before the reader unnoticed and unrecognized." In a trice our mind ran back to our school days, and we thought how our quondam tutor—Scotch and given to whisky—would have enunciated "Pontifex Ma a a r x i moos." Still farther skipped our fancy, and as we walked up Genesee street we forgot the Dutch signs, and the lager-bier, and the smell of sour-krout from out the doors, and we stood in the Rome of ancient days. Past the vast coliseum moved a procession of patrician mothers, bearing in their arms the infant scions of Rome's noblest families. Palms crowned their heads, and in advance walked white robed priests of solemn mien. The temple was reached. Here bending with their offerings before the pure white altars, were mothers yet to be, laden with their coming maternity; while the band of fruitful ones whom we accompanied walked grandly up toward the lofty altar, where sat enthroned—an infant on her lap and in her hand a diaper—the marble statue of the Goddess Cloaci—— *No! a Diis infernis! Lucina!*

O egregious blunder! O miserable euphony! which passed beneath so many careful eyes still undetected. Poor Mrs. Malaprop and Sister Partington! "The allegory on the banks of the Nile" of the one, or the "I would make them cry copaiva! copaiva!" of the other, are things that need our sympathy and not deserve our sneers.

Genesee St. savored more strongly than ever of sour-krout as we walked meekly along among the wood-sawyers. H.

---

Reese's National Medical Gazette has, in its March number, a long and elaborate article on Inhalation in Diseases of the Lungs, by Dr. Robert Hunter; accompanied by a decided editorial endorsement of the writer. We had been led, from reading Dr. Hunter's advertisements in the daily prints, to set him down as an empiric, but Dr. Reese's endorsement places the matter in a different light, as it is impossible that he should have been ignorant of Dr. Hunter's claims to professional standing. H.

